# Enhanced expression of *OsNAC5* leads to up-regulation of *OsNAC6* and changes rice (*Oryza sativa* L.) ionome

**DOI:** 10.1590/1678-4685-GMB-2022-0190

**Published:** 2023-05-05

**Authors:** Andriele Wairich, Ariane Vitali, Janete Mariza Adamski, Karina Letícia Lopes, Guilherme Leitão Duarte, Lucas Roani Ponte, Henrique Keller Costa, Paloma Koprovski Menguer, Rinaldo Pires dos Santos, Janette Palma Fett, Raul Antonio Sperotto, Felipe Klein Ricachenevsky

**Affiliations:** 1 Universidade Federal do Rio Grande do Sul, Centro de Biotecnologia, Programa de Pós-Graduação em Biologia Celular e Molecular (PPGBCM), Porto Alegre, RS, Brazil.; 2 Universidade Federal do Rio Grande do Sul, Instituto de Biociências, Departamento de Botânica, Porto Alegre, RS, Brazil.; 3 Universidade Federal de Santa Maria, Instituto de Ciências Naturais e Exatas, Departamento de Biologia, Porto Alegre, RS, Brazil.; 4 Universidade do Vale do Taquari (Univates), Programa de Pós-Graduação em Biotecnologia (PPGBiotec), Lajeado, RS, Brazil.; 5 Universidade Federal de Pelotas, Programa de Pós-Graduação em Fisiologia Vegetal (PPGFV), Pelotas, RS, Brazil.

**Keywords:** Oryza sativa, NAC, iron, zinc, stress

## Abstract

NAC transcription factors are plant-specific proteins involved in many processes during the plant life cycle and responses to biotic and abiotic stresses. Previous studies have shown that stress-induced *OsNAC5* from rice (*Oryza sativa* L.) is up-regulated by senescence and might be involved in control of iron (Fe) and zinc (Zn) concentrations in rice seeds. Aiming a better understanding of the role of *OsNAC5* in rice plants, we investigated a mutant line carrying a T-DNA insertion in the promoter of *OsNAC5*, which resulted in enhanced expression of the transcription factor. Plants with OsNAC5 enhanced expression were shorter at the seedling stage and had reduced yield at maturity. In addition, we evaluated the expression level of *OsNAC6*, which is co-expressed with *OsNAC5*, and found that enhanced expression of *OsNAC5* leads to increased expression of *OsNAC6*, suggesting that *OsNAC5* might regulate *OsNAC6* expression. Ionomic analysis of leaves and seeds from the *OsNAC5* enhanced expression line revealed lower Fe and Zn concentrations in leaves and higher Fe concentrations in seeds than in WT plants, further suggesting that OsNAC5 may be involved in regulating the ionome in rice plants. Our work shows that fine-tuning of transcription factors is key when aiming at crop improvement.

## Introduction

Rice (*Oryza sativa* L.) is one of the three most important crops in the world, being the staple food for over three billion people (Ali and Wani 2021). However, rice plants often suffer from a variety of biotic and abiotic stresses, such as mineral imbalance, salt, drought, cold, high temperature, pathogens, and phytophagous pests. These biotic and abiotic stresses directly or indirectly affect plant growth and development and may decrease crop yield (Ali *et al*., 2021). To cope with stress, plants have evolved a series of defence mechanisms, which commonly include transcription factors (TFs) controlling downstream genes that code for proteins involved in stress response, acclimation, and tolerance. 

The NAC protein family comprises plant-specific TFs that are characterized by the presence of a highly conserved N-terminal DNA binding domain (NAC domain), as well as non-conserved C-terminal domains (Ernst et al., 2004; Olsen et al., 2005; Fang et al., 2008). NAC is an acronym derived from three proteins containing the domain: NAM (*no apical meristem*), ATAF1,2 (*Arabidopsis transcription activator factor*) and CUC2 (*cup-shaped cotyledon*) (Souer et al., 1996; Aida et al., 1997). The NAC proteins have been implicated in transcriptional control of a variety of plant processes, including responses to phytohormones and to stresses (Meng et al., 2007; Sperotto *et al*., 2009; Jeong et al., 2010, 2013; Kim et al., 2013; Ricachenevsky et al., 2013; Lee et al., 2017; Liu et al., 2020; Li *et al*., 2021). The rice genome has 151 genes predicted to encode members of the NAC TF family (Nuruzzaman et al., 2010). Rice NACs are classified into five groups (I-V), and the most well-characterized is sub-group III, also known as stress-responsive NAC (SNAC) (Fang *et al*., 2008). Members of this group are majorly involved in stress responses, and some genes already had their functional role characterized (Nakashima et al., 2007; Pyung et al., 2007; Hu et al., 2008; Sperotto *et al*., 2009; Zheng et al., 2009; Jeong *et al*., 2010; Takasaki et al., 2010; Song et al., 2011).

Given their role in stress response, NAC TFs were already used to improve stress tolerance in engineered rice plants. Overexpression of *SNAC1*, *OsNAC9*, *OsNAC10* and *OsNAC109* led to increased tolerance to various abiotic stresses including drought, salinity and low temperature, as well as changes in plant architecture, seed set and senescence (Hu et al., 2006; Jeong et al., 2010; Redillas et al., 2012; Li et al., 2021). In another study, rice plants overexpressing *OsNAC6* were tolerant to abiotic and biotic stresses such as drought, salinity and blast disease (Nakashima et al., 2007; Lee et al., 2017), whereas *osnac6* loss-of-function mutant plants were susceptible to drought (Lee *et al*., 2017). Curiously, *OsNAC6* overexpression also leads to a short plant phenotype (Nakashima *et al*., 2007).

Senescence can be induced by exogenous factors such as phytohormones, nutrient availability and environmental stresses (Sperotto *et al*., 2009; Ricachenevsky et al., 2013; Lee and Masclaux-Daubresse 2021). Senescence is a form of programmed cell death, which is tightly coordinated at the organism, cellular and molecular levels. During senescence, organelles and macromolecules are disassembled and nutrients and metabolites are remobilized through the vascular system from source tissues to young leaves or reproductive organs (Yoshida 2003; Sperotto *et al*., 2009; Ricachenevsky *et al*., 2013). 

Previously, it was shown that *OsNAC5* encodes an abscisic acid (ABA)-responsive TF up-regulated by natural and induced senescence processes (Sperotto *et al*., 2009). Comparison of four rice cultivars revealed that *OsNAC5* up-regulation is higher and earlier in flag leaves and panicles of IR75862 plants, which have higher seed concentrations of iron (Fe), zinc (Zn) and protein than the other three cultivars, suggesting a role of OsNAC5 on remobilization of nutrients from green tissues to seeds (Sperotto *et al*., 2009). In wheat, expression of the *NAM-B1* gene (*OsNAC5* ortholog), an ancestral allele of a NAC TF, is responsible for the earlier onset of flag leaf senescence, resulting in more efficient remobilization of protein, Zn, Fe and Mn from leaves to the grains (Uauy et al., 2006; Distelfeld et al., 2007). *OsNAC5* gene has also been related to Fe-deficiency responses in rice plants (Ogo et al., 2006), besides being speculated that *OsNAC5* has a role in senescence and metal movement to rice grains by controlling, either directly or indirectly, the biosynthesis of the metal chelator nicotianamine (NA) and metal transport through the phloem (Ricachenevsky et al., 2013). Interestingly, OsNAC6 up-regulates the expression of genes involved in NA biosynthesis (*OsNAS1* and *OsNAS2*), promoting the accumulation of NA (Lee et al., 2017), which could lead to Fe and Zn mobilization and accumulation in rice seeds (Lee *et al*., 2009). It was also found that OsNAC5 protein binds to *OsNAS1* promoter, likely up-regulating *OsNAS1* expression (Chung et al., 2018). Altogether, these data suggest stress-related NAC TFs might be involved in regulating the ionome of rice plants. 

*OsNAC5* is also up-regulated by abiotic stresses such as high salinity, drought, low-temperature, methyl jasmonate (MeJA) and ABA (Takasaki et al., 2010). OsNAC5 interacts with other stress-regulated NAC proteins such as OsNAC6, SNAC1, as well as itself, forming homodimers and heterodimers (Jeong et al., 2009). *OsNAC5*-overexpressing rice plants had improved tolerance to high salinity (Takasaki *et al*., 2010), whereas silencing of *OsNAC5* decreased tolerance to cold, drought and salt stress (Song et al., 2011). In addition, root-specific overexpression of *OsNAC5* led to enlarged roots and conferred enhanced drought tolerance and increased grain yield under greenhouse conditions (Jeong *et al*., 2013). Therefore, it is clear that NAC TFs can be useful for stress-tolerance improvement, but it is necessary to fine-tune their expression. 

To better understand the role of *OsNAC5* in rice plants, in this work we investigated a rice mutant line carrying a T-DNA insertion in the promoter of *OsNAC5*. We found that the T-DNA insertion caused increased expression of *OsNAC5*, resulting in decreased growth and reduced yield. Plants with enhanced expression of *OsNAC5* also showed increased expression of *OsNAC6*, suggesting that OsNAC5 might positively regulate *OsNAC6* expression, likely by an indirect mechanism. The mutant line presented decreased Fe and Zn concentrations in leaves and increased Fe concentration in seeds, suggesting that OsNAC5 is involved in regulating the ionome of rice plants. Our data indicate that using *OsNAC5* to generate stress tolerant plants needs fine-tuning of expression levels to avoid possible deleterious effects, and that stress-related NACs might regulate each other. 

## Material and Methods

### Plant materials and treatments

A T-DNA line (PFG_1D-03641) with an insertion at 496 bp upstream from the *OsNAC5* (Os11g0184900/LOC_Os11g08210) transcription start site (based on the mRNA sequence XM_015761800.2) and 604 bp from the translation start site (based on the coding sequence of the locus ID LOC_Os11g08210) was retrieved from the Pohang University of Science and Technology (POSTECH) Seed Bank. The T-DNA line was generated in Hwayoung (HWA) wild-type (hereafter “WT”) background, and all comparisons were performed in relation to WT plants. Primers suggested by iSect Primer tool were used to confirm the presence of the T-DNA insertion. The vector used for generating the T-DNA insertion line was described previously (Jeong et al., 2002). Briefly, our promoter insertion line was produced using vector pGA0727. The vector has a Tubulin A1 promoter, Tubulin A1 second intron, HPT hygromycin resistance gene and Tubulin A1 terminator close to the left border; and promoterless GUS gene followed by the NOS terminator (Jeong *et al*., 2002). 

Rice seeds were germinated and cultivated in laboratory conditions, as described by Wairich et al. (2019). Briefly, seeds were sown in petri dishes with filter paper soaked in distilled water. Germinated seeds were transferred to plastic containers with plant holders adapted as lids, and were cultivated in hydroponic media containing 700 μM K_2_SO_4_, 100 μM KCl, 100 μM KH_2_PO_4_, 2 mM Ca(NO_3_)_2_, 500 μM MgSO_4_, 10 μM H_3_BO_3_, 0.5 μM MnSO_4_, 0.5 μM ZnSO_4_, 0.2 μM CuSO_4_, 0.01 μM (NH_4_)_6_Mo_7_O_24_, and 100 μM Fe^+3^-EDTA, pH 5.4 (Ricachenevsky et al., 2011). Nutrient solution was changed every 3-4 days, and plants were kept under 25 ± 2 ºC, photoperiod 16/8 hours light/dark. Measurements of shoot and root length were taken 20 days after germination (n = 10-12 plants per genotype). 

For hormonal treatments, 30-day-old rice plants grown as described above were sprayed with 10 µM of ABA, 10 µM of MeJA, or 10 mM of ethrel (an ethylene precursor), and harvested after 1, 2 or 3 hours. For experiments with plants at the reproductive stage, samples were collected from field-grown rice plants, as described in Sperotto *et al*. (2009). Briefly, we used the Counce et al. (2000) scale to collect samples from flag leaves and developing panicles: R3 (panicle exertion), R5 (grain filling) and R7 (grain maturation). Plant height and agronomical traits associated with yield as panicles per plant, total seeds per panicle and per plant, seed length and weight of 1,000 full seeds were recorded at harvest. 

Dark induced senescence experiments and ABA/Benzyl-Amino Purine (BAP) treatments were conducted as described by Sperotto *et al*. (2009) and Ricachenevsky et al. (2010). Briefly, 2 cm^2^ leaf sections were soaked in MES buffer in a 24 well plate containing either only MES, 50 µM of ABA or 50 µM of BAP. Plates were kept in the dark wrapped in aluminium foil for seven days. RNA extractions were performed using 8-10 leaf sections per samples, with n = 3 sample in total. 

All experiments, unless otherwise stated, were conducted using *Oryza sativa* cv. Nipponbare. 

### RNA extraction and gene expression analyses

Total RNA was extracted from harvested plant tissues using the Concert Plant RNA Reagent (Invitrogen^®^, Carlsbad, USA), following treatment with DNase I (Life Technologies^®^, Carlsbad, USA). First strand cDNA was prepared using M-MLV Reverse Transcriptase (Life Technologies) and 1 μg of total RNA, according to the manufacturer’s instructions. All primers (listed in Table S1) were designed to amplify 100-150 bp of the 3’-UTR of the genes and to have similar Tm values (60 ± 2 °C). Reaction settings were composed of an initial denaturation step of 5 min at 94 °C, followed by 40 cycles of 10 s at 94 °C, 15 s at 60 °C, 15 s at 72 °C; samples were held for 2 min at 60 °C for annealing of the amplified products and then heated from 60 to 99 °C with a ramp of 0.3 °C/s to provide the denaturing curve of the amplified products. Reactions contained 10 μl of 100 times diluted cDNA, 2 μl of 10X PCR buffer, 1.2 μl of 50 mM MgCl_2_, 0.1 μl of 5 mM dNTPs, 0.4 μl of 10 μM primer pairs, 4.25 μl of water, 2.0 μl of SYBR green (1:10,000, Molecular Probe), and 0.05 μl of Platinum *Taq* DNA polymerase (5 U/μl, Invitrogen^®^), in 20 μl final volume. Data were analyzed using the comparative Ct (threshold cycle) method (Livak and Schmittgen 2001). The PCR efficiency was obtained for each individual amplification plot using the LinRegPCR software (Ramakers et al., 2003). In each plate, the average of PCR efficiency for each amplicon was determined and used in further calculations. Ct values for all genes were normalized to the Ct value of the rice ubiquitin gene *UBQ5* (Jain et al., 2006). The equation Q0 target gene/Q0 *UBQ5* = [(Eff *UBQ5*)^Ct UBQ5^ / (Eff target gene)^Ct target gene^], where Q0 corresponds to the initial amount of transcripts, was used for normalization. Each data point corresponds to three true biological replicate samples, each of them evaluated in four technical replicates.

### Anatomical measurements

To assess the areas (μm^2^) of aerenchyma, intercellular space in the aerenchyma and vascular system in leaf sheath of the WT and T-DNA mutant with enhanced expression (hereafter "OsNAC5-EX") plants (30-day-old plants), leaf sheath fragments of the third completely expanded leaf were collected (n = 7 per genotype). Samples were fixed in a mixture of 1% glutaraldehyde and 4% formaldehyde in 0.1 M phosphate buffer for 24 h (McDowell and Trump 1976) and dehydrated using a graded ethanol series. Subsequently, leaf sheath samples were infiltrated and embedded in 2-hydroxyethyl methacrylate-based resin (Gerrits and Smid 1983). The 4 µm cross sections made with a rotary microtome (Leica Microm HM 340E) were stained with 0.1% (w/v) toluidine blue O (C.I. 52040) in phosphate buffer aqueous solution (pH 6.8). Images were obtained with a Leica DMRB bright field light microscope, equipped with digital color camera (Leica DFC500). Area measurements were calculated using Zeiss software (Axiovision Rel. 4.8). 

### Leaf and seeds elemental analyses by inductively coupled plasma - mass spectrometry (ICP-MS)

Elemental concentration analyses of leaf and seeds samples were performed as described by Ricachenevsky et al. (2018), adapted for rice samples. Plants from WT and *OsNAC5-EX* were cultivated in hydroponics with nutrient solution, as described above, for 37 days. The third fully expanded leaf was collected for analyses (n = 6). In addition, the elemental concentration was evaluated in seeds of WT and *OsNAC5-EX* rice plants grown until maturity in the greenhouse. Three seeds per genotype were employed for the elemental analysis.

### Co-expression analysis of *OsNAC5*

To examine the co-expression pattern of *OsNAC5*, a gene network search was performed using a ‘guide gene approach’, in which a single guide gene (Os11g0184900/LOC_Os11g08210) was employed to explore other functionally related genes, using the online RiceFREND plataform (Sato et al., 2013). A gene network, which consists of the *OsNAC5* gene and the genes connected to it, is presented in Figure S1. 

### Statistical analysis

Mean values were compared by One-Way ANOVA followed by Tukey test (*p <* 0.05) or Student’s *t* test (*p* < 0.5, 0.1, and 0.01), using the GraphPad Software (http://graphpad.com/quickcalcs/ttest2/).

## Results

### Identification of a T-DNA line overexpressing *OsNAC5*

**Figure 1 -:**
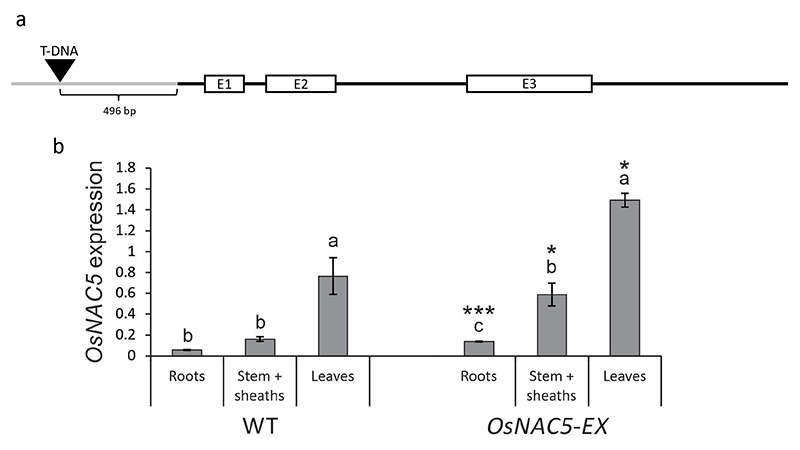
Identification of an *OsNAC5* enhanced expression (*OsNAC5-EX*) line. (a) Gene structure and T-DNA insertion site in the promoter region of *OsNAC5*. Exons are shown as boxes (E1, E2, E3); introns, 5’UTR and 3’UTR are shown as black bars; promoter region is shown as a grey bar; T-DNA insertion site is depicted by a triangle. (b) The relative transcript levels of *OsNAC5* in roots, stem + sheaths and leaves in Hwayoung (WT) and *OsNAC5-EX* genotypes (n = 10-12). Data presented are means ± SE. Different letters above the bars indicate significant differences (*P-*value<0.05; post-hoc Tukey’s test) among tissues in the same genotype. Asterisks indicate statistical differences comparing the same tissue between different genotypes (Student *t*-test, *P-value < 0.05, ***P-value < 0.001).

To examine the physiological function of *OsNAC5*, a mutant line bearing a single T-DNA fragment inserted 604 bp upstream of the translation initiation site of the *OsNAC5* gene (Figure 1A) was analysed. Expression analyses, performed by RT-qPCR in roots, stem + sheaths and leaves, showed that this line has enhanced levels of *OsNAC5* expression compared with WT plants. Leaves had the most pronounced difference comparing the mutant line and WT, followed by stem + sheaths and roots (Figure 1B). These results indicated that the insertion enhances expression of *OsNAC5* rather than disrupting it. Therefore, we consider this line an *OsNAC5* enhanced expression (EX) line (hereafter *OsNAC5-EX*). 

### *OsNAC5-EX* plants have decreased growth at the seedling stage

**Figure 2 -:**
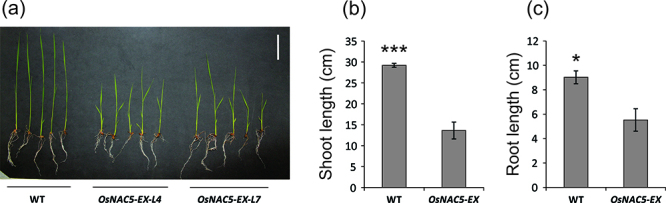
Phenotypic analyses of *OsNAC5-EX*. (a) Twenty-day-old plants grown in hydroponic solution. (b) Shoot length (cm) (n= 10-12). (c) Root length (cm) (n= 10-12). Data represent means ± SE. Asterisks indicate statistical differences comparing Hwayoung (WT) and *OsNAC5-EX* plants (Student *t*-test, *P-value < 0.05, ***P-value < 0.001).

When cultivated in hydroponic solution for 20 days, homozygous lines *OsNAC5-EX-L4* and *OsNAC5-EX-L7* (two independently segregating, homozygous lines derived from the same heterozygous insertional mutant line) showed clear phenotypic differences compared to WT plants (Figure 2A). Both roots and shoots lengths are smaller compared to WT (Figure 2B-C). These results suggest that the enhanced expression of *OsNAC5* impairs plant growth at the seedling stage.

**Figure 3 -:**
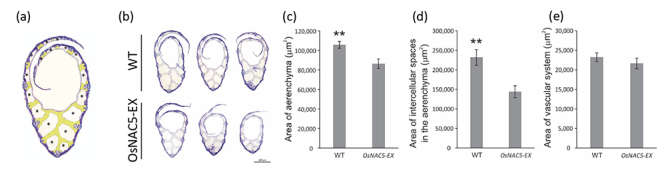
Anatomical analysis showing the variations in the morphology of leaf sheaths of WT and *OsNAC5-EX*. (a) Schematic view of anatomical measurements. We used the second WT image from (b). Yellow areas show the aerenchyma cells and asterisks show the intercellular spaces from aerenchyma. (b) Photomicrographs of leaf sheath cross sections of Hwayoung (WT) and *OsNAC5-EX* plants*;* (c) Area of aerenchyma (µm^2^); (d) Area of intercellular spaces in the aerenchyma (µm^2^); (e) Area of vascular system (µm^2^) from Hwayoung (WT) and *OsNAC5-EX* plants (n = 7 plants). Data represent means ± SE. Asterisks indicate statistical differences comparing Hwayoung (WT) and *OsNAC5-EX* plants (Student *t*-test, **P-value < 0.01).

To understand size differences, we characterized the leaf anatomy of *OsNAC5-EX* and WT with emphasis on the more clearly visible tissue of the leaf sheath: the aerenchyma. We performed semi-thin section analysis of leaf sheath of the mutant line and WT plants (Figure 3A) and found that the aerenchyma area (corresponding to aerenchyma cells) and the intercellular spaces of the aerenchyma (which correspond only to the air spaces) were smaller in *OsNAC5-EX* than in WT plants (Figure 3A-C). However, we found no difference in the area of vascular system between *OsNAC5-EX* and WT plants (Figure 3D). These results demonstrate that the mutation does not change the structure of the vascular system but decreases total area of aerenchyma tissue and air spaces, which shows that leaf length in *OsNAC5-EX* appears to be the result of the tissue changes (cellular and extracellular dimensions) in the ground system or mesophyll.

### Yield components are low in *OsNAC5-EX* plants

**Figure 4 -:**
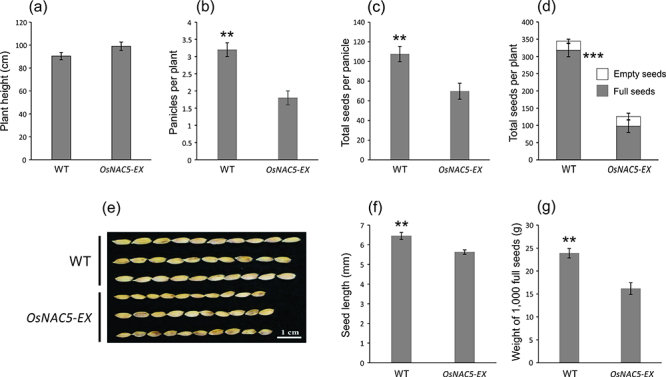
Enhanced expression of *OsNAC5* impairs yield components. (a) Plant height (cm); (b) Panicles per plant; (c) Total seeds per panicle; (d) Total seeds per plant; (e) Morphology of 10 grains harvested from three independent plants; (f) Seed length (mm); (g) Weight of 1,000 full seeds from Hwayoung (WT) and *OsNAC5-EX* plants (n = 5). Data represent means ± SE. Asterisks indicate statistical differences comparing Hwayoung (WT) and *OsNAC5-EX* plants (Student *t*-test, **P-value < 0.01, ***P-value < 0.001).

To investigate the effects of enhanced expression of *OsNAC5* in yield, we examined several agronomic traits in *OsNAC5-EX* and WT plants at the harvest stage. Despite the decreased growth observed in *OsNAC5-EX* plants at the seedling stage, no difference in plant height was observed at maturity (Figure 4A). However, yield attributes were lower in *OsNAC5-EX* plants than in WT plants, including panicles per plant, total seeds per panicle and total full seeds per plant (Figure 4B-D). No difference was observed in total empty seeds per plant comparing the genotypes (Figure 4D). Moreover, *OsNAC5-EX* plants produced smaller grains than WT (Figure 4E-F), resulting in reduced weight per 1,000 full seeds (Figure 4G). Overall, grain yield per plant was impaired in *OsNAC5-EX* plants. 

### Enhanced expression of *OsNAC5* decreases the concentrations of leaf essential nutrients

The *OsNAC5* gene was previously identified in a suppression subtractive hybridization analysis from flag leaves of IR75862 plants, a rice cultivar with high Fe, Zn and protein concentrations in seeds (Sperotto *et al*., 2009). Elemental analyses were performed on leaves of WT and *OsNAC5-EX* plants under greenhouse condition to evaluate whether the enhanced expression of *OsNAC5* alters the leaf ionome. The concentrations of potassium (K) and arsenic (As) from *OsNAC5-EX* plants were higher than the concentration observed in WT plants (Table 1). On the other hand, the enhanced expression of *OsNAC5* led to a significant reduction in the concentrations of magnesium (Mg), calcium (Ca), manganese (Mn), Fe, Zn and molybdenum (Mo). These results indicate that the enhanced expression of *OsNAC5* leads to perturbations in the leaf ionome. The concentration of some macroelements as phosphorus (P) and sulphur (S) did not change with enhanced expression of *OsNAC5* gene (Table 1). 

**Table 1 -:** Concentrations of Mg, P, S, K, Ca, Mn, Fe, Co, Ni, Cu, Zn, As, Mo and Cd in leaves of WT and *OsNAC5-EX* plants cultivated in hydroponics with nutrient solution.

Element	WT	*OsNAC5-EX*
**Mg**	6320.79 ± 0.28*	5014.67 ± 0.32
**P**	3466.66 ± 168.47	3822.76 ± 313.25
**S**	4935.02 ± 346.95	5318.61 ± 196.56
**K**	14718.85 ± 0.85	17956.06 ± 0.94*
**Ca**	9266.35 ± 0.70*	6977.86 ± 0.57
**Mn**	786.38 ± 44.08**	586.17 ± 34.13
**Fe**	61.59 ± 3.56*	49.03 ± 4.19
**Co**	0.061 ± 0.005	0.074 ± 0.004
**Ni**	0.68 ± 0.03	0.72 ± 0.06
**Cu**	7.08 ± 0.56	7.43 ± 0.38
**Zn**	28.69 ± 1.68*	21.30 ± 0.99
**As**	0.069 ± 0.003	0.105 ± 0.012*
**Mo**	2.85 ± 0.16***	1.46 ± 0.12
**Cd**	0.0182 ± 0.00073	0.0220 ± 0.0048

Data are means ± standard errors. *n* = 6. Mean values indicated by one, two or three asterisks are different by the Student's *t* test (*P* ≤ 0.05, 0.01, and 0.001, respectively).

Concentrations are presented as mg.g^-1^ DW. DW = dry weight.

### Enhanced expression of *OsNAC5* changes the rice seed ionome

To investigate the possible role of *OsNAC5* on the remobilization of mineral nutrients from green tissues to grains, analyses of 14 element were performed on seeds of WT and *OsNAC5-EX* plants cultivated under greenhouse condition. Seeds from *OsNAC5-EX* plants contained higher concentrations of essential elements for human nutrition than WT seeds (Table 2). Among the elements that had increased concentrations in *OsNAC5-EX* seeds, we highlight Mg, P, S, K and, especially, Fe. Potassium concentration in *OsNAC5-EX* seeds was approximately 100% higher than the concentration in WT seeds. In addition, the Fe concentration increased more than 4 µg.g^-1^ DW in seeds from the *OsNAC5-EX* line. These results further indicate a role of *OsNAC5* in the regulation of the seed ionome. 

**Table 2 -:** Concentrations of Mg, P, S, K, Ca, Mn, Fe, Co, Ni, Cu, Zn, As, Mo, and Cd in seeds of WT and *OsNAC5-EX* plants cultivated under greenhouse condition.

Element	WT	*OsNAC5-EX*
**Mg**	915.09 ± 39.73	1092.89 ± 12.65*
**P**	2362.19 ± 123.34	3882.27 ± 150.16**
**S**	1495.75 ± 45.52	1965.00 ± 37.05**
**K**	5547.10 ± 34.38	11332.50 ± 1398.58*
**Ca**	178.59 ± 2.62	212.13 ± 24.88
**Mn**	80.29 ± 3.67	76.53 ± 5.05
**Fe**	13.29 ± 0.97	18.00 ± 0.73*
**Co**	0.07 ± 0.009	0.10 ± 0.01
**Ni**	0.62 ± 0.06	0.96 ± 0.13
**Cu**	5.63 ± 0.44	8.70 ± 0.37**
**Zn**	34.69 ± 0.52	38.64 ± 3.95
**As**	0.17 ± 0.01	0.20 ± 0.02
**Mo**	0.20 ± 0.01	0.28 ± 0.01*
**Cd**	0.0162 ± 0.003	0.02 ± 0.007

Data are means ± standard errors. *n* = 3. Mean values indicated by one or two asterisks are different by the Student's *t* test (*P* ≤ 0.05 and 0.01, respectively). Concentrations are presented as mg.g^-1^ DW. DW = dry weight.

### *OsNAC5* is co-expressed with *OsNAC6*

Aiming to understand which genes might be functionally associated with *OsNAC5*, we performed a co-expression analysis (Figure S1 and Table S2). Interestingly, another gene encoding a NAC TF (*OsNAC6* - Os01g0884300) is co-expressed with *OsNAC5*, being induced by various stress conditions in rice plants (Ohnishi et al., 2005; Nakashima et al., 2007; Lee et al., 2017). It is noteworthy that *OsNAC6* overexpression also resulted in a short plant phenotype during the vegetative stage (Nakashima *et al*., 2007; Takasaki et al., 2010).

We also found that *OsNAC5* is co-expressed with the ATP-dependent zinc metalloprotease FTSH 5 (Os01g0574500), which binds one zinc ion per subunit. A zinc finger protein from the RING/FYVE/PHD-type (Os04g0417400), identified as up-regulated in leaves of rice seedlings (*O*. *sativa* cv. Nipponbare) after four days of Fe excess treatment (Finatto et al., 2015) was also co-expressed with OsNAC5, as well as a 2OG-Fe(II) oxygenase domain containing protein previously identified as up-regulated by Fe excess in rice leaves and recently suggested as an Fe sensor during altered Fe availability (Bashir et al., 2014). 

### Enhanced expression of *OsNAC5* affects *OsNAC6* expression

**Figure 5 -:**
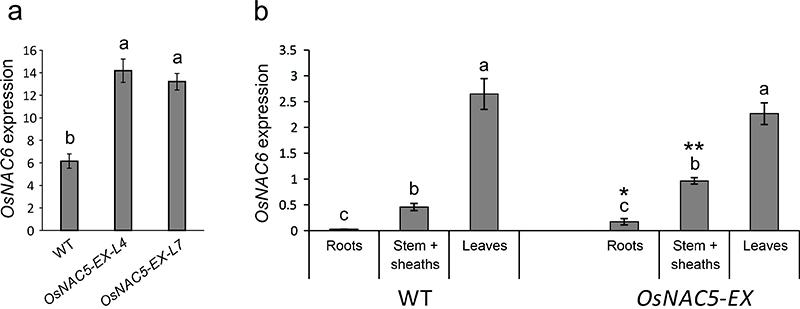
Enhanced expression of *OsNAC5* influences *OsNAC6* expression. (a) Relative transcript levels of *OsNAC6* in shoots of Hwayoung (WT) plants and two homozygous lines with enhanced expression of *OsNAC5* (*OsNAC5-EX-L4* and *OsNAC5-EX-L7*) grown under control condition for 20 days. (b) Relative transcript levels of *OsNAC6* in three different tissues during the vegetative development in Hwayoung (WT) and *OsNAC5-EX* genotypes. Gene expression data is relative to rice *Ubiquitin 5* expression. Data represent means ± SE (three biological replicates, four technical replicates per sample). Different letters above the bars indicate significant differences (*P-*value<0.05; post-hoc Tukey’s test) among plant organs in the same genotype. Asterisks indicate statistical differences comparing the same organs in different genotypes (Student *t*-test, *P-value < 0.05, **P-value < 0.01).

*OsNAC5* shares 82.5% identity with *OsNAC6* (Ooka et al., 2003). Both are induced by drought, salt, and ABA treatments (Hu et al., 2006, 2008; Nakashima et al., 2007; Jeong et al., 2013), and both proteins can physically interact as a heterodimer (Jeong *et al*., 2009). Our co-expression analysis also suggests a functional relationship between the two genes (Figure S1). We also noticed that the *OsNAC5-EX* phenotype (Figure 2A) resembled that of *OsNAC6* overexpressing plants (Nakashima *et al*., 2007). Therefore, we hypothesized that *OsNAC5*-*EX* might show altered *OsNAC6* gene expression. To test such hypothesis, we conducted RT-qPCR of *OsNAC6* expression in whole shoots of 20 days old plants from WT and *OsNAC5-EX* lines. The expression of *OsNAC6* was significantly higher in both *OsNAC5-EX* lines than in WT plants (Figure 5A). 

Furthermore, the expression of *OsNAC6* was evaluated in three different organs during vegetative development of WT and *OsNAC5-EX* plants. *OsNAC6* expression was evidently higher in leaves, compared to roots and stem + sheaths of WT plants. However, besides the higher expression of *OsNAC6* in roots and stem + sheaths of *OsNAC5-EX* lines than in WT, such difference was not observed in leaves (Figure 5B). This could be explained by the high expression level of *OsNAC6* in leaves. Still, our data confirm that *OsNAC5* enhanced expression leads to increased *OsNAC6* expression, which might therefore contribute to the short plant phenotype.

### Expression of *OsNAC6* during vegetative and reproductive stages closely resemble that of *OsNAC5*

**Figure 6 -:**
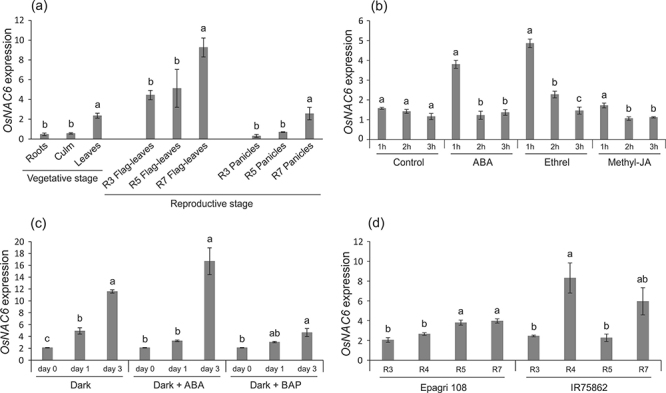
Relative expression levels of *OsNAC6* in (a) rice Nipponbare plants grown under control condition during vegetative and reproductive stages; (b) Leaves of Nipponbare plants grown under control condition and sprayed with 10 µM of abscisic acid (ABA), 10 µM of methyl-jasmonate, and 10 mM of ethrel and harvested after 1, 2 and 3 hours after spraying; (c) Leaves of Nipponbare plants grown under dark, dark + ABA, and dark + BAP treatment and harvested 0, 1 and 3 days after the onset of the treatment; (d) Flag leaf during age-induced senescence of Epagri 108 and IR75862 cultivars. Gene expression data is relative to rice *Ubiquitin 5* expression. Data represent means ± SE (three biological replicates, four technical replicates per sample). Different letters above the bars indicate significant differences (*P-*value<0.05; post-hoc Tukey’s test) among tissues in the same treatment in different times.

Given the possible functional relationship between OsNAC5 and OsNAC6, we conducted RT-qPCR analyses of *OsNAC6* expression during vegetative and reproductive stages in rice plants from the Nipponbare genotype, using the same experimental conditions previously described for analysing *OsNAC5* expression (Sperotto *et al*., 2009). During the vegetative stage, *OsNAC6* expression was clearly higher in leaves, compared to roots and stem + sheath, although detected in all organs (Figure 6A). During the reproductive stage, *OsNAC6* expression in flag leaves was already high at R3, steadily increasing towards maturation, reaching the maximum level at R7 (Figure 6A). In panicles, *OsNAC6* transcripts also accumulate during maturation, although the initial and final expression levels are lower than in flag leaves. These results indicate that *OsNAC6* expression increases during seed maturation and senescence in reproductive tissues. Thus, the pattern observed for *OsNAC6* closely matches the pattern previously found for *OsNAC5* (Sperotto *et al*., 2009), further suggesting that both genes might regulate each other, either directly or indirectly. 

### *OsNAC6* expression is regulated by ABA and ethylene

Short-term expression analyses after spraying rice leaves with ABA, Me-JA or Ethrel (which is converted to ethylene in plants) showed that *OsNAC6* expression increases 2.4- and 3.0- fold after one hour of ABA and Ethrel treatments, respectively (Figure 6B). Interestingly, three hours after treatment, *OsNAC6* expression in Ethrel-treated plants were still higher than control (1.56 fold), but expression levels in ABA-treated plants were similar to control. Expression levels after six hours of treatment were comparable to control in both treatments. No differences were observed in Me-JA treated plants (Figure 6B). These results show that *OsNAC6* is responsive to ABA and ethylene. 

### *OsNAC6* is a senescence-associated gene

Although leaf senescence occurs in an age-dependent manner, the initiation and progression of senescence can be induced by a variety of plant hormones and stress conditions (such as ABA and dark) by increasing the expression of several senescence associated genes (SAG) (Sperotto *et al*., 2009; Li et al., 2021). Our group has previously suggested that *OsNAC5* is a SAG induced by ABA, possibly involved in the senescence process and in nutrient remobilization from flag leaves to developing grains (Sperotto *et al*., 2009). To test whether *OsNAC6* is also a SAG, we submitted detached leaves to dark-induced senescence, dark + ABA-induced senescence, and dark + BAP (senescence delayed condition; Sperotto *et al*., 2009; Ricachenevsky et al., 2010). When rice leaves were placed in the dark, *OsNAC6* transcripts steadily increased (Figure 6C). When ABA was added for three days, *OsNAC6* expression achieved the highest level detected in this experiment. On the other hand, when BAP was added for three days, transcript accumulation was subtle and did not reach the high levels observed in dark or dark + ABA conditions (Figure 6C). These results show that *OsNAC6* is indeed a SAG, with an expression pattern similar to *OsNAC5* (Sperotto *et al*., 2009).

### Differential *OsNAC6* expression in flag leaf during age-induced senescence in cultivars with contrasting levels of Fe, Zn and protein in grains

Previously, we have shown that *OsNAC5* expression in flag leaves during age-induced senescence is correlated to final Fe, Zn and protein concentrations in grains of several rice cultivars (Sperotto *et al*., 2009). As *OsNAC6* expression is highly correlated with *OsNAC5*, we hypothesized that *OsNAC6* expression could be similar in flag leaves. Therefore, we quantified transcript accumulation in flag leaves of two rice cultivars with low (EPAGRI 108) and high (IR75862) concentrations of Fe, Zn and protein in the grains. Transcripts of *OsNAC6* presented higher and similar levels at R5 and R7 stages in the EPAGRI 108 cultivar. On the other hand, IR75862 cultivar presented the highest levels of *OsNAC6* expression at R4 and R7 stages (Figure 6D), a pattern resembling that observed previously for *OsNAC5* (Sperotto *et al*., 2009). These results suggest that *OsNAC6* could also be involved in age-induced senescence and nutrient remobilization, as proposed for *OsNAC5* (Sperotto *et al*., 2009; Sperotto *et al*., 2010; Ricachenevsky et al., 2013). 

## Discussion

NAC proteins belong to a plant-specific family of TF with 117 and 151 members in Arabidopsis and rice genomes, respectively. In rice, this family is divided into five groups according to phylogenetic relationships (Fang et al., 2008; Nuruzzaman et al., 2010). Several members from the NAC TF family are involved in plant growth and development, leaf senescence, grain filling, metal homeostasis and tolerance to biotic and abiotic stresses, such as drought, cold and salinity (Ohnishi et al., 2005; Nakashima et al., 2007, 2012; Redillas et al., 2012; Jeong et al., 2013; Lee et al., 2017; Sharma et al., 2019; Mathew et al., 2020; Yan et al., 2021; Li et al., 2021). This study has evaluated a T-DNA insertion line in which *OsNAC5* expression was enhanced. Our results showed that this line (*OsNAC5-EX*) expresses increased levels of *OsNAC5* especially in shoots (Figure 1B). Higher expression of *OsNAC5* in shoots than in roots was observed by Sperotto *et al*. (2009) and Song et al. (2011) when evaluating WT plants under control condition. This suggests that the plants characterized here might have enhanced *OsNAC5* expression in a pattern that resembles that of the native promoter. That may explain differences from lines overexpressing *OsNAC5* under the control of constitutive promoters. 

Another important caveat that needs to be highlighted is that our work is based on a single T-DNA insertion line. Given the well-known effects of rice tissue culture and transformation on the genome integrity (a.k.a. somaclonal variation; Miyao et al., 2012), it is common practice in the field to have more than one mutant/overexpression line for gene characterization. However, the promoter insertion found in our work cannot be easily compared to other T-DNA-generated lines, since insertion in the same position would not be feasible, and insertions along the promoter, but in different positions could have different effects. To partially circumvent this problem, we found two homozygous plants segregating from the same heterozygous line identified at first. Of course, we should still consider that some variation might be fixed in the line and could contribute to the phenotypes observed. Importantly, we should point out that knockout lines would not be a proper comparison, and even overexpression lines (Song et al., 2011) would be different, since in overexpression lines, the native gene maintains its expression domains, developmental timing, cell-specificity, etc, with the transgene adding to the overall expression level. It is possible that our line’s phenotype is derived from the changes in *OsNAC5* locus, which result in changes in expression level as well minute changes in developmental timing, cell type, tissue, etc, rather than only increased expression level overall. Our data therefore should be interpreted considering this caveat.

Lower shoot and root growth were observed in *OsNAC5-EX* seedlings than in WT (Figure 2). Our results are contrasting with the results observed by Song et al. (2011), which found no phenotypical differences between *OsNAC5* overexpressing and WT lines under control conditions. The impairment of growth observed in *OsNAC5-EX* could be a consequence of the overexpression of stress-related genes, which is often associated with an impairment on growth and leads to productivity loss. Similar situation was observed in Arabidopsis plants overexpressing the gene *DREB1A* under the control of 35S promoter (*35S::DREB1A*), which displays growth retardation and a severe reduction in seed production (Liu et al., 1998; Kasuga et al., 1999), and in transgenic rice lines expressing *35S::OsNAC6*, which exhibited decreased growth, abnormal development and reduced seed production (Nakashima et al., 2007). 

The enhanced expression of *OsNAC5* caused reduction in yield components. Similar growth retardation and low yields were also observed when *OsNAC10* was expressed under the control of a constitutive (GOS2) promoter (*GOS2::OsNAC10*) (Jeong et al., 2010), and in transgenic rice plants constitutively overexpressing *OsNAC6* (Nakashima et al., 2007). These results highlight that the ectopic expression of a stress response gene is not always a straightforward, effective strategy to achieve stress tolerance, leading to growth abnormalities and yield penalties. In this way, a more effective strategy when overexpressing a TF is the employment of tissue-specific promoters, especially when aiming at the fine-tuning of genes associated with a specific developmental stage or with a reproductive organ (Jeong *et al*., 2010). 

Previous studies have shown that *OsNAC5* was induced by a number of abiotic stresses, such as drought, natural (aging) and induced (dark) senescence, cold and salt (Sperotto *et al*., 2009). A high and early *OsNAC5* expression was observed in flag leaves (R4 stage) and panicles of IR75862 plants, a rice cultivar with high seed concentrations of Fe, Zn and protein (Sperotto *et al*., 2009). In addition, seed Fe and Zn concentrations were positively correlated with *OsNAC5* expression in flag leaves during R3 (Sperotto *et al*., 2010). These findings are in accordance with the ones described here, in which plants of *OsNAC5-EX* showed a reduction in the concentration of essential elements such as Mg, Fe and Zn in leaves when compared to WT plants (Table 1). Such decrease in nutrient concentration may indicate that plants with enhanced expression of *OsNAC5* have a higher translocation from these nutrients from leaves to other organs such as seeds via phloem. In addition, the increase of Mg, P, S, K and Fe in seeds (Table 2) corroborate with the higher translocation of nutrients from leaves to seeds in *OsNAC5-EX* plants. These results could explain the alteration in the leaf and seed ionomes observed in the *OsNAC5-EX* genotype.

*OsNAC6* (*SNAC2*) also belongs to the SNAC subfamily in rice (Fang et al., 2008). As observed for *OsNAC5*, the expression of *OsNAC6* is induced by various biotic and abiotic stresses including wounding, blast disease, cold, drought, high salinity, JA and ABA (Ohnishi et al., 2005; Nakashima et al., 2007; Hu et al., 2008). A co-expression analysis, which is an indicator of functional correlation between genes, showed that *OsNAC6* is co-expressed with *OsNAC5* (Figure S1 and Table S2). Together with previous reports demonstrating that OsNAC5 forms both homo- and heterodimers with other stress-associated NAC proteins, such as OsNAC6 and SNAC1 (Jeong et al., 2009; Takasaki et al., 2010), these data suggest that OsNAC5 could regulate *OsNAC6* expression, either directly or indirectly. ChIP-Seq analyses did not find OsNAC5 or OsNAC6 binding to each other’s promoter (Chung et al., 2018), thus suggesting an indirect regulation. In addition, our results also found genes associated with leaf senescence, development and Fe excess in the co-expression network (Bashir et al., 2014; Finatto et al., 2015). These results point out for a possible role of OsNAC5 on Fe homeostasis. Furthermore, *OsNAC5* was induced by Fe excess treatment in *O*. *sativa* and wild rice, *O*. *meridionalis* (Wairich et al., 2021). Previous work also showed that OsNAC5 binds to OsNAS1 promoter (Chung *et al*., 2018) and that OsNAC6 regulates NA accumulation in rice plants (Lee et al., 2017). However, the role of OsNAC5 on Fe homeostasis needs more in-depth studies. It would be interesting to specifically evaluate Fe homeostasis in *OsNAC5* and *OsNAC6*-overexpressing plants (either constitutively or in roots; Jeong *et al*., 2013; Lee *et al*., 2017) described in the literature to further test these hypotheses.

Senescence is the last stage of leaf development, and plays an important role in crop yield and nutritional quality, as nutrients are relocated from senescent tissues to sink organs, as grains (Tong et al., 2021). When evaluating the expression profile of *OsNAC6* during vegetative and reproductive stages, we observed increased expression of *OsNAC6* during the reproductive stage, especially in R7 panicles and flag leaves, which represent seed maturation and leaf senescence, respectively (Figure 6A). This result is in accordance with the ones previously reported for *OsNAC5* (Sperotto *et al*., 2009) and for *OsNAC6* (Nakashima et al., 2007).

The role of OsNAC6 as an ABA-dependent TF was confirmed by a significant increase in *OsNAC6* expression in detached leaves incubated in dark + ABA, which accelerates the senescence process (Figure 6C). A similar expression pattern was observed for the *OsNAC5* gene when rice plants are submitted to salt stress-inducing ABA-mediated senescence (Sperotto *et al*., 2009). In addition, a few other NAC TFs are involved in regulating age induced senescence, such as ONAC16 (Sakuraba et al., 2015), OsNAC2 (Mao et al., 2017), OsNAP (Liang et al., 2014) and OsNAC109 (Li *et al*., 2021). Furthermore, OsNAC10 is also associated with leaf senescence and increases nutrient mobilization from leaves to developing seeds, playing a key role in rice grain filling (Sharma et al., 2019). Therefore, more attention should be paid to the putative role of NAC TFs, especially OsNAC5 and OsNAC6, as regulators of senescence and nutrient remobilization processes. Future work should address whether these two TFs regulate each other, and in which organs or tissues they may act synergistically. The nature of this co-regulation is unlikely to be direct (Chung et al., 2018) and deserves further attention. 

*OsNAC5* was identified as responsive to ABA, Me-JA and other plant hormones such as ethylene, auxin, SA and brassinolide (Sperotto *et al*., 2009; Jeong et al., 2010; Takasaki et al., 2010; Song et al., 2011), and ABA is likely to be involved in regulating *OsNAC5*-dependent induction of tolerance to abiotic stress (Song *et al*., 2011). *OsNAC6* is also induced by cold, drought, high salinity and ABA application (Ohnishi et al., 2005; Nakashima et al., 2007, 2012). To further support a functional relationship between *OsNAC5* and *OsNAC6*, we evaluated the *OsNAC6* short-term transcriptional changes in response to ABA, ethylene (using Ethrel) and Me-JA. *OsNAC6* was only induced by ABA and ethylene (Figure 6B). ABA is a plant hormone which is involved in regulating a plethora of processes associated with plant growth and development, such as seed dormancy and germination, leaf senescence, seedling growth and other process. It is considered a stress hormone, being regulated by both biotic and abiotic stresses (Sun et al., 2020). We speculate that the tolerance phenotype conferred by *OsNAC6* (Nakashima *et al*., 2007; Lee et al., 2017), in addition to the role on leaf senescence, could be ABA- and/or ethylene-dependent. Further work is needed to explore such hypotheses.

## Conclusion

Processes related to plant development, such as flowering and senescence, have direct effects on cereal yield and nutritional quality (Alptekin et al., 2021). In addition, biotic and abiotic stresses adversely affect plant growth and productivity. In this context, a better elucidation of target genes regulating responses to stresses and leaf senescence has the potential for advancing the productivity and nutritional quality of cereal grains. The potential overexpression of NAC genes, especially *OsNAC5* as previously proposed, aiming at high tolerance and improvement in grain yield, should be fine-tuned to avoid deleterious effects. Our results suggest that *OsNAC6* expression follows the same pattern as observed for *OsNAC5*, raising the possibility that *OsNAC6* is involved in the same regulatory network, although the mechanism for such co-regulation is not known. Furthermore, this work suggests a role of OsNAC5 and OsNAC6 proteins regulating ABA-dependent leaf senescence, and suggests a role of OsNAC5 on leaf and seed ionomes as well as on Fe remobilization from leaves to grains. We speculate that these processes might be linked, but further in-depth work on both transcription factors is needed to test this hypothesis.

## Supplementary material

The following online material is available for this article:

Table S1 - Gene-specific PCR primers used for qRT-PCR.

Table S2 - Genes co-expressed with *OsNAC5*.

Figure S1 - Gene network showing *OsNAC5* (Os11g0184900) and co-expressed/connected genes.
