# Supplementary material for: Enhanced expression of OsNAC5 leads to up-regulation of OsNAC6 and changes rice (Oryza sativa L.) ionome
Source: Genet Mol Biol. 2023 May 5;46(1 Suppl 1):e20220190. doi: 10.1590/1678-4685-GMB-2022-0190 (PMC10161346; doi:10.1590/1678-4685-GMB-2022-0190)
Supplement: Table S1 - [file 1415-4757-GMB-46-1-s1-e20220190-s1.pdf]

Genetics and Molecular Biology
https://doi.org/10.1590/1678-4685-GMB-2022-0190

Supplementary Material to "Enhanced expression of *OsNAC5* leads to up-regulation of *OsNAC6* and changes rice (*Oryza sativa* L.) ionome"

Table S1 - Gene-specific PCR primers used for qRT-PCR.

Gene	Forward primer 5' → 3'	Reverse primer 5' → 3'
OsNAC5	CAGCAGCTGATGGTATTGTC	AGAGACCTGTTTGGCACGAA
OsNAC6	CAGCAGAAAGATGGTGCAAT	AGGAAAGCTAGCCCCCTGTA
OsUBQ5	ACCACTTCGACCGCCACTACT	ACGCCTAAGCCTGCTGGTT
