# Supplementary material for: Enhanced expression of OsNAC5 leads to up-regulation of OsNAC6 and changes rice (Oryza sativa L.) ionome
Source: Genet Mol Biol. 2023 May 5;46(1 Suppl 1):e20220190. doi: 10.1590/1678-4685-GMB-2022-0190 (PMC10161346; doi:10.1590/1678-4685-GMB-2022-0190)
Supplement: Table S2 - [file 1415-4757-GMB-46-1-s1-e20220190-s2.pdf]

Genetics and Molecular Biology
https://doi.org/10.1590/1678-4685-GMB-2022-0190

Supplementary Material to "Enhanced expression of *OsNAC5* leads to up-regulation of *OsNAC6* and changes rice (*Oryza sativa* L.) ionome"

Table S2 - Genes co-expressed with *OsNAC5*.

Hierarchy	LocusID	Description
0	Os11g0184900	Similar to NAC-domain protein 5-7
1	Os01g0191700	Similar to Pyrophosphate-fructose-6-phosphate 1-phosphotransferase-like protein (Pyrophosphate-dependent phosphofructo-1-kinase-like protein)
1	Os01g0201200	Similar to Protein kinase
1	Os01g0574500	Peptidase M41, FtsH domain containing protein
1	Os01g0842200	Similar to Scarecrow-like 9 (Fragment)
1	Os01g0884300	No apical meristem (NAM) protein domain containing protein
1	Os01g0933500	Conserved hypothetical protein
1	Os02g0259100	Conserved hypothetical protein
1	Os03g0723700	Conserved hypothetical protein
1	Os04g0417400	U box domain containing protein
1	Os05g0429000	Similar to Hydroxymethyltransferase
1	Os05g0458400	Similar to AAA-metalloprotease FtsH
1	Os05g0566200	Similar to Small CTD phosphatase 1 splice variant
1	Os07g0647200	Cytochrome P450 family protein
1	Os08g0392100	2OG-Fe(II) oxygenase domain containing protein
1	Os08g0460600	Conserved hypothetical protein
1	Os08g0468100	Nitrate reductase [NADH] 1 (EC 1.7.1.1) (NR1)
1	Os08g0468700	Similar to Nitrate reductase [NADH] 1 (EC 1.7.1.1) (NR1)
1	Os10g0529700	Similar to Glutathione S-transferase GST 35 (EC 2.5.1.18)
1	Os11g0155600	Similar to Multidrug-resistance associated protein 3
1	Os11g0536000	Conserved hypothetical protein
1	Os12g0100500	Alpha/beta hydrolase family protein
