# Supplementary material for: Enhanced expression of OsNAC5 leads to up-regulation of OsNAC6 and changes rice (Oryza sativa L.) ionome
Source: Genet Mol Biol. 2023 May 5;46(1 Suppl 1):e20220190. doi: 10.1590/1678-4685-GMB-2022-0190 (PMC10161346; doi:10.1590/1678-4685-GMB-2022-0190)
Supplement: Figure S1 - [file 1415-4757-GMB-46-1-s1-e20220190-s3.pdf]

Genetics and Molecular Biology
https://doi.org/10.1590/1678-4685-GMB-2022-0190

Supplementary Material to "Enhanced expression of *OsNAC5* leads to up-regulation of *OsNAC6* and changes rice (*Oryza sativa* L.) ionome"

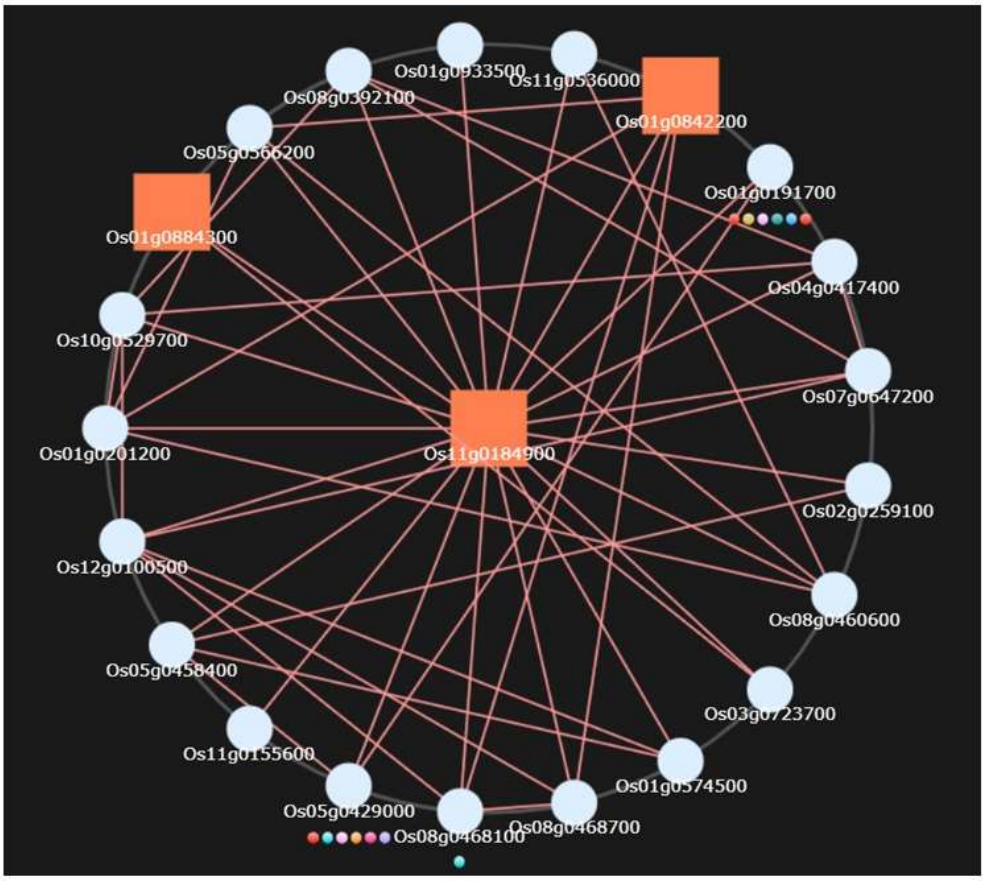

Figure S1 - Gene network showing *OsNAC5* (Os11g0184900) and co-expressed/connected genes.
